# CAPABLE CARE PARTNER

**DOI:** 10.1093/geroni/igae098.0586

**Published:** 2024-12-31

**Authors:** Deborah Paone, Jeanne Schuller, Sarah Szanton

**Affiliations:** Johns Hopkins University, Baltimore, Maryland, United States; Paone & Associates, LLC, Minneapolis, Minnesota, United States; Johns Hopkins University, Baltimore, Maryland, United States

## Abstract

We created and tested a prototype of CAPABLE Care Partner as an enhancement to CAPABLE. We developed this through a three-phased approach over three years and incorporated care partners, older adults, clinicians, and administrators to design and test this enhancement using human-centered design principles. We created this to recognize the role and supportive actions of care partners and to provide some support for their needs as well as those of the older adult participant. We conducted a pilot in 2023 in three U.S. implementation sites--programs that had successfully offered CAPABLE for at least one year. Results from post-pilot assessments and 360 degree qualitative interviews showed positive effect on care partners, and provided real-world insights on how to incorporate a social support specialist into the program, so that the care partner and the older adult dyad connect to social support/community resources, attend to self-care, and are better positioned to address current and future needs to age in community.

